# Urothelial neoplasm of the bladder in childhood and adolescence: a rare disease

**DOI:** 10.1590/S1677-5538.IBJU.2015.0200

**Published:** 2016

**Authors:** Haci Polat, Mehmet M. Utangac, Murat T. Gulpinar, Ali Cift, Ibrahim Halil Erdogdu, Gul Turkcu

**Affiliations:** Adiyaman University, Faculty of Medicine, Department of Urology, Adiyaman, Turkey; Dicle University, Faculty of Medicine, Department of Urology, Diyarbakir, Turkey; Canakkale Onsekiz Mart University, Faculty of Medicine, Department of Urology, Canakkale, Turkey; Adiyaman University, Faculty of Medicine, Department of Medical Pathology, Adiyaman, Turkey; Dicle University, Faculty of Medicine, Department of Medical Pathology, Diyarbakir, Turkey

**Keywords:** Urinary Bladder Neoplasms, Adolescent, Ultrasonography

## Abstract

**Purpose:**

Bladder tumors are rare in children and adolescents. For this reason, the diagnosis is sometimes delayed in pediatric patients. We aimed to describe the diagnosis, treatment, and follow-up methods of bladder urothelial neoplasms in children and adolescents.

**Materials and Methods:**

We carried out a retrospective multicenter study involving patients who were treated between 2008 and 2014. Eleven patients aged younger than 18 years were enrolled in the study. In all the patients, a bladder tumor was diagnosed using ultrasonography and was treated through transurethral resection of the bladder (TURBT).

**Results:**

Nine of the 11 patients (82%) were admitted with gross hematuria. The average delay in diagnosis was 3 months (range, 0–16 months) until the ultrasonographic diagnosis was performed from the first episodes of macroscopic hematuria. A single exophytic tumor (1–4cm) was present in each patient. The pathology of all patients was reported as superficial urothelial neoplasm: two with papilloma, one with papillary urothelial neoplasm of low malignant potential (PUNLMP), four with low grade pTa, and four with low grade pT1. No recurrence was observed during regular cystoscopic and ultrasonographic follow-up.

**Conclusions:**

Regardless of the presence of hematuria, bladder tumors in children are usually not considered because urothelial carcinoma in this population is extremely rare, which causes a delay in diagnosis. Fortunately, the disease has a good prognosis and recurrences are infrequent. Cystoscopy may be unnecessary in the follow-up of children with bladder tumors. We believe that ultrasonography is sufficient in follow-up.

## INTRODUCTION

Bladder cancer (BC) is the most common malignancy of the urinary tract. It is the seventh most common cancer in men and the seventeenth most common cancer in women. The worldwide age-standardized incidence rate is 9 per 100.000 for men and 2 per 100.000 for women (2008 data) (1). Despite these high rates in the general population, it is very rare in the pediatric age group. According to a recent study, approximately 110 cases have been reported in the literature in this age group since 1950 (2). These tumors have a low grade of malignancy, showing little tendency toward recurrence and have a good prognosis. Bladder tumors are not usually considered in children and adolescents, even in the presence of hematuria, because they are extremely rare. We aimed to draw our colleague's attention to this issue by conducting a multicenter study (three centers) covering data from 11 patients who were diagnosed as having this disease over the last seven years, because we have treated four patients for bladder urothelial neoplasm in our clinic in the last three years.

We aimed to describe the presentation, diagnostic methods, treatment, pathologic examination, and follow-up methods of bladder urothelial neoplasms in the pediatric age group.

## MATERIALS AND METHODS

In the period from 2008 to 2014, we identified 11 urothelial bladder tumors in patients aged younger than 18 years by means of a retrospective multicenter (three centers) study.

A detailed history was taken from all patients and their parents. The patients were specifically questioned about whether they had any of the known risk factors for bladder cancer. Due to the planning of this study, all 11 patients were contacted and questioning was repeated. The initial diagnosis of bladder tumor was confirmed by ultrasonography. After the initial diagnosis, each of the patients underwent transurethral resection of bladder tumor (TURBT) for a definitive diagnosis and treatment. None of the patients received a repeat TURBT and none of the patients received additional intracavitary chemotherapy or immunotherapy. Similarly, each patient's cystoscopy and ultrasonography were reviewed every six months during the first year and once a year in subsequent years of the follow-up.

## RESULTS

The characteristics of the patients are described in Table-1. The main symptoms of the patients, delayed diagnosis time, and the tumor location and characteristics are also shown in Table-1.

**Table 1:** Patients' characteristics.

Age/Sex	Reason for application	The risk factors for bladder cancer	Delayed time for diagnosis (months)	Diagnostic tool	Location on bladder	The appearance of the tumor	Pathological condition (WHO/ISUP)	Recurrence	Follow-up (years)
15/M	Hematuria	None	16	USG	Trigone	Single, papillary, 4cm	PUNLMP	No	2
16/F	Hematuria	None	3	USG	Lateral wall	Single papillary, 1cm	Papillary-LG, Ta	No	3
17/F	Hematuria	None	None	USG	Lateral wall	Single, papillary, 2–3 cm	Papillary-LG, T1	No	1
17/M	Hematuria	Paint workmanship (4 years); smoking (3 years)	1	USG	Lateral wall	Single papillary, 3cm	Papillary-LG, Ta	No	3
12/M	Hematuria	None	1	USG	Lateral wall	Single papillary, 2 cm	Papillary-LG, Ta	No	1.5
17/M	Hematuria	Smoking (2 years)	4	USG	Trigone	Single papillary, 4 cm	Papillary-LG, T1	No	7
12/M	Abdominal pain	None	None	USG	Lateral wall and trigone	Single, papillary, 2–3 cm	Papilloma	No	6
17/F	Hematuria	None	2	USG	Lateral wall and trigone	Single, papillary, 4cm	Papillary-LG, T1	No	1
16/M	Incidental	None	None	USG	Lateral wall	Single papillary, 1–2 cm	Papillary-LG, Ta	No	2
17/F	Hematuria	None	4	USG	Lateral wall	Single, papillary, 4 cm	Papillary-LG, T1	No	5
17/F	Hematuria	None	2	USG	Trigone	Single, papillary, 2 cm	Papilloma	No	2.5

**ISUP** = International Society of Urological Pathology; **Papillary-LG** = papillary urothelial carcinoma, low grade; **PUNLMP** = papillary urothelial neoplasm of low malignant potential; **USG** = Ultrasonography; **WHO** = World Health Organization;

Nine of the 11 patients were admitted with one or more episodes of gross hematuria and one patient was admitted with abdominal pain. A diagnosis had been made at least one month after the first episode of hematuria in eight of the 11 patients. In our patients, the diagnosis had been made at an average of 3 months (0–16 months) after the first hematuria episode. When the reasons for this delay were investigated, different causes were shown to have affected the patients: two patients reported presenting late to the hospital; four patients cited a delay in investigation, and two patients said there had been a combination of both these reasons. Only three patients (27%) in our study had been diagnosed on time.

The patients were questioned about the known risk factors for bladder tumors in adults. Exposure to paint products (4 years) and smoking (3 years) were risk factors identified in one patient, and smoking (2 years) was a risk factor identified in one other patient. No risk factors were detected in the other nine patients.

**Figure 1:**
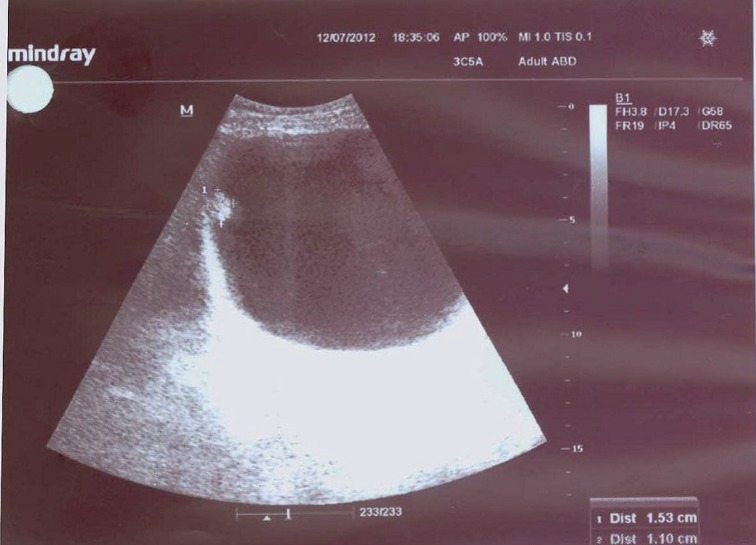
Bladder ultrasonography showing the soft-tissue lesion in the right bladder wall of a girl aged 17-years.

**Figure 2:**
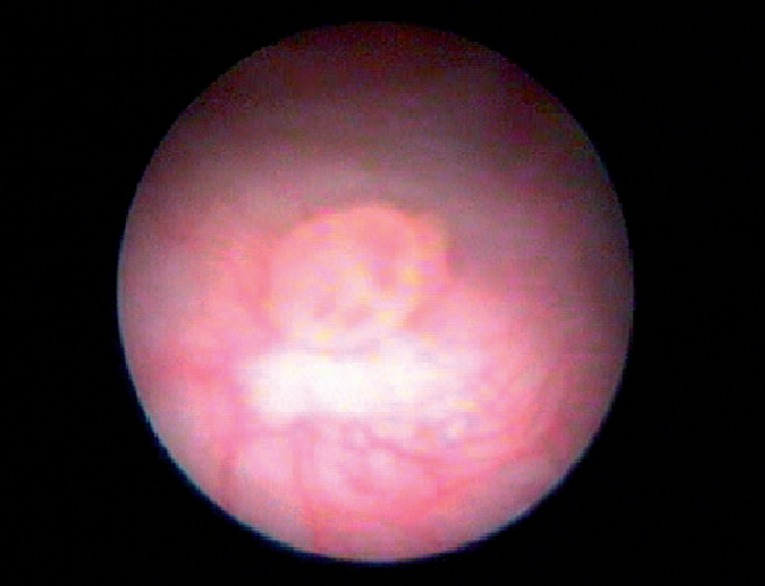
Cystoscopic view of a papillary bladder tumor (AP: low grade Ta).

**Figure 3:**
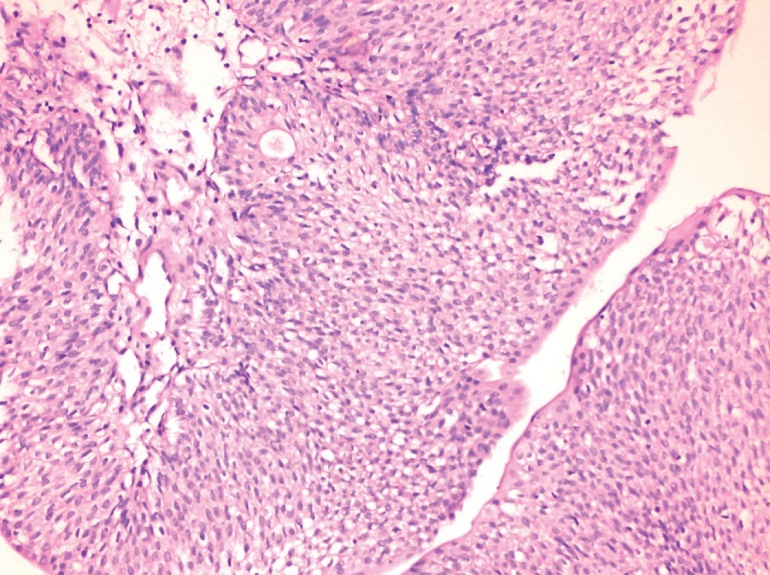
Histologic examination showing mild pleomorphism in cells and uncertain nucleoli consistent with low-grade papillary urothelial carcinoma, pT1 (H&E, × 200).

All of the patients tumors were initially diagnosed using ultrasonography. Diagnostic cystoscopy before transurethral resection was not performed in any of the patients. After ultrasonography examination, TURBT was performed under general anesthesia. A single papillary lesion was identified in all patients; the tumor was located either in the lateral wall or in the trigone region of the bladder. The size of the lesions ranged from 1–4cm. The pathology of all patients was reported as superficial urothelial neoplasm in accordance with the World Health Organization/International Society of Urological Pathology (WHO/ISUP) 2004 histologic classification (3); two patients were found to have papilloma, one had papillary urothelial neoplasm of low malignant potential (PUNLMP), four had low grade pTa, and four had low grade pT1 (Figures 1–3).

The mean follow-up time for the patients was 3 years (range, 1–7 years). Follow-up was performed with cystoscopy and ultrasonography. The cystoscopy was reviewed every six months during the first year and once a year in subsequent years. At the same schedule the patients were examined using ultrasonography. Relapse was not observed on ultrasonography or cystoscopy in any of the 11 patients. Two of the 11 patients in our study underwent several cytology examinations but at irregular intervals; however, they were reported to be negative for the presence of a tumor. No recurrence was found among the patients at the average 3-year (range, 1–7 years) follow-up.

## DISCUSSION

Although bladder cancer is seen in all ages, it is extremely rare in younger patients, especially in children and adolescents. Therefore, when bladder cancer is seen in this age group, its etiologic factors and prognosis become the focus of attention. Unfortunately, as in our study, the diagnosis is sometimes delayed, probably because of the rarity of this diagnosis and the predominance of benign causes of hematuria in this age group (4). Fortunately, bladder tumors are often seen as superficial and low grade (I–II) with a low malignant potential in children and adolescents. In our study, one patient was diagnosed 16 months after the first painless gross hematuria. The patients and their parents were questioned again for this study; three patients with hematuria including this patient said that their hematuria had improved after being admitted to hospital.

In our study, hematuria was the main diagnostic symptom in nine of the eleven patients (82%). One patient's cancer was diagnosed with ultrasonography while being examined because of abdominal pain. Similar to our results, the literature describes the presence of gross hematuria in 80–100% of all cases (2, 5). All patients in our study had a single papillary tumor in the lateral wall or trigone region of the bladder. After complete TURBT, none of the patients received additional intracavitary chemotherapy or immunotherapy. We believe that additional intracavitary therapy after TURBT may be unnecessary in these papillary tumors owing to the rarity of recurrence and because these types of tumors are not associated with progression to invasive disease. For the same reasons, we did not behave eager for a second resection. However, none of the patients had recurrence of bladder tumor.

For pediatric patients, the best follow-up methods after TURBT procedures are still the subject of debate in the literature. Surveillance methods in pediatric patients with bladder urothelial neoplasm have not been described in the literature. The recurrence of bladder tumors in pediatric patients is very low, recurrent tumors are benign, and the lesions are low grade. In their series of 23 patients, Fine et al. described a 13% recurrence, as compared with the adult rate of 40–70% (6).

According to our current opinion, when we consider the results of our patients together with data in the literature, cystoscopy may have been unnecessary and should not be performed for follow-up in children and adolescents with bladder tumors. In pediatric patients, ultrasonography at certain intervals may be sufficient for follow-up. In a study with a small series that supports our suggestion, Hoenig et al. found that ultrasonography was extremely effective in identifying bladder tumors, and the authors argued for its use in initial diagnosis and disease surveillance (5). In our study, bladder tumors were detected in the first ultrasonography in all of the patients. Moreover, no tumors were detected in any patient in the follow-up ultrasonography, which is compatible with the cystoscopy findings.

## CONCLUSIONS

Although bladder tumors are rare in children and adolescents, it should be considered in the presence of painless gross hematuria. In this study, we recognized that urothelial bladder tumors in children and adolescents have a good prognosis owing to the low malignancy and the low rate of recurrence. No recurrence was observed in our patients. As a result, it is likely that we performed redundant cystoscopies in the follow-up of these patients. We suggest that ultrasonography should be used as the follow-up method. If a suspected tumor is detected on ultrasonography, it might be sufficient to perform cystoscopy at that time. It is clear that there is a need to conduct a study using a larger series in order to arrive at a definite conclusion as to whether ultrasonography alone is sufficient; however, a study of a large case series does not seem likely.

## ABBREVIATIONS

ISUP: = International Society of Urological Pathology
Papillary-LG: = Papillary urothelial carcinoma, low grade
PUNLMP: = Papillary urothelial neoplasm of low malignant potential
TURBT: = Transurethral resection of the bladder
USG: = Ultrasonography
WHO: = World Health Organization
